# The Central Noradrenergic System in Neurodevelopmental Disorders: Merging Experimental and Clinical Evidence

**DOI:** 10.3390/ijms24065805

**Published:** 2023-03-18

**Authors:** Alessandro Galgani, Emanuele Bartolini, Marta D’Amora, Ugo Faraguna, Filippo Sean Giorgi

**Affiliations:** 1 Department of Translational Research and of New Surgical and Medical Technologies, University of Pisa, 56126 Pisa, Italy; alessandro.galgani@phd.unipi.it (A.G.);; 2 Department of Developmental Neuroscience, IRCCS Fondazione Stella Maris, 56128 Pisa, Italy; 3 Tuscany PhD Programme in Neurosciences, 50121 Florence, Italy; 4 Department of Biology, University of Pisa, 56125 Pisa, Italy; 5 Istituto Italiano di Tecnologia, 16163 Genova, Italy

**Keywords:** locus coeruleus, noradrenaline, neurodevelopment, developmental disorders, autism, ADHD, neuropediatric, neurogenesis, childhood epilepsy

## Abstract

The aim of this article is to highlight the potential role of the locus-coeruleus–noradrenergic (LC-NA) system in neurodevelopmental disorders (NdDs). The LC is the main brain noradrenergic nucleus, key in the regulation of arousal, attention, and stress response, and its early maturation and sensitivity to perinatal damage make it an interesting target for translational research. Clinical data shows the involvement of the LC-NA system in several NdDs, suggesting a pathogenetic role in the development of such disorders. In this context, a new neuroimaging tool, LC Magnetic Resonance Imaging (MRI), has been developed to visualize the LC in vivo and assess its integrity, which could be a valuable tool for exploring morphological alterations in NdD in vivo in humans. New animal models may be used to test the contribution of the LC-NA system to the pathogenic pathways of NdD and to evaluate the efficacy of NA-targeting drugs. In this narrative review, we provide an overview of how the LC-NA system may represent a common pathophysiological and pathogenic mechanism in NdD and a reliable target for symptomatic and disease-modifying drugs. Further research is needed to fully understand the interplay between the LC-NA system and NdD.

## 1. Introduction

The central noradrenergic system plays crucial roles in maintaining the homeostasis of the adult brain and influences various neural networks that control cognition and behavior. The main source of noradrenaline (NA) in the human brain is the locus coeruleus (LC), a pontine nucleus that supplies NA to the entire central nervous system (CNS), except for the basal ganglia. In recent years, the involvement of this nucleus in adult neurological disorders has been increasingly recognized, not only thanks to sophisticated neuropathological postmortem analysis in patients and several experimental models, but also to advances in neuroimaging tools that eventually enabled the in vivo study of LC integrity [1]. These findings have led to the hypothesis that LC impairment may play a key role in neurodegenerative disorders (namely, Parkinson’s disease, and Alzheimer’s disease), in parallel with further emphasizing the significance of this nucleus in CNS physiology. These findings prompt further investigation into the LC-NA system in other medical conditions and raise questions about what changes might occur in the LC during infantile disorders and how these changes may impact the pathophysiology and occurrence of neurodevelopmental diseases (NdD). According to the Diagnostic and Statistical Manual of Mental Disorders, Fifth Edition, Text Revision (DSM-5-TR), NdD are characterized by developmental deficits or differences in brain processes that produce impairment of personal, social, academic, or occupational functioning. NdDs comprise intellectual disability (ID), communication disorders, autism spectrum disorder (ASD); attention-deficit/hyperactivity disorder (ADHD), neurodevelopmental motor disorders—including tic disorders—and specific learning disorders [2].

In this review, we aim to provide a general perspective on the LC-NA system in neurodevelopment and infancy, framing the current physiological knowledge within the clinical spectrum of NdD. In particular, an overview of the following aspects will be provided: (a) LC ontogenesis; (b) the role of LC in neurodevelopment; (c) clinical evidence and experimental findings supporting LC-NA impairment in NdD. Then, we will discuss the possible pathogenic role of LC involvement based on the abovementioned points and eventually propose future experimental and clinical studies that may profit from innovative in vivo tools and animal models.

## 2. Methods

**Figure 1:**
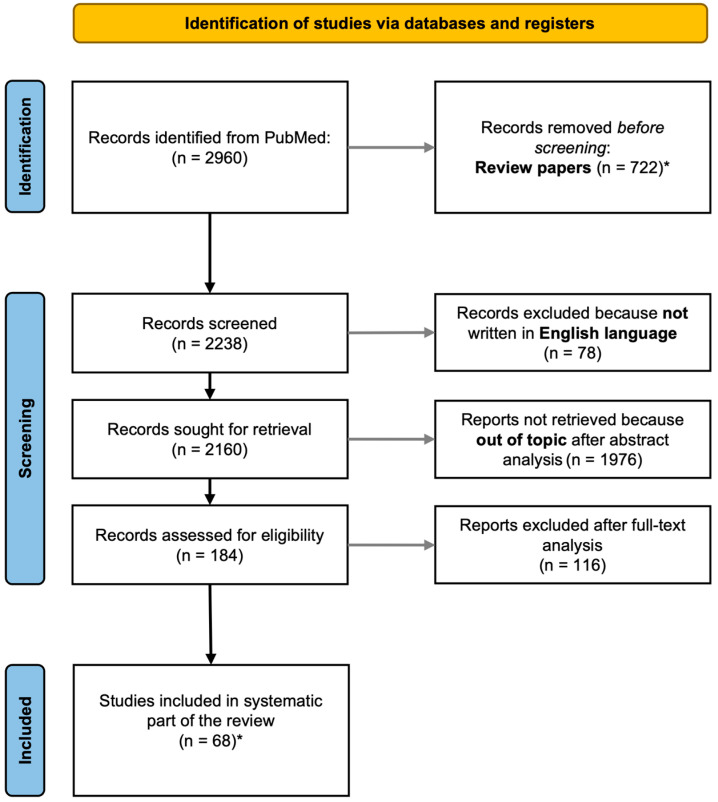
The workflow of the literature search is reported in the flowchart. The search was performed on pubmed.ncbi.nlm.nih.gov using the key words reported in the paragraph “Methods”. A final set of 68 papers were used to write the systematic part of the review. * Articles cited as [3,4,5,6,7,8,9,10] are review papers used to provide a clinical and pharmacological background and are thus not included in the final count.

We performed a two-stage literature search using the pubmed.ncbi.nlm.nih.gov search platform. In the first stage, we searched for papers describing LC ontogeny and its involvement in CNS development, using the following keywords: “locus coeruleus”, “ontogeny”, “development”, “brain development”, and “embryogenesis.” In the second stage, we searched for papers reporting data on the involvement of the LC-NA system in NdDs; the keywords used were “locus coeruleus,” “norepinephrine,” “neurodevelopmental disorders,” “autism spectrum disorders,” “ADHD,” and “childhood neurological disorders.” We included only original articles written in English. Figure 1 shows the flow chart of the literature search. The review of collected studies was performed by three of the authors (AG; EB; FSG). The selected papers were used to write the descriptive sections of the review (Section 4, Section 5, Section 6, Section 7 and Section 8), which report the results of the systematic review of the literature. The introductory sections (Section 1 and Section 2) and discussion sections (Section 9 and Section 10) were written by referring also to original articles, review papers, and book chapters authored by prominent researchers in the field of LC-NA system physiology and pathology. It should be mentioned right away that, concerning clinical articles, only studies about ASD and ADHD were found. The review is not registered, and no review protocol has been prepared.

## 3. LC Anatomy and Basic Description of Its Functions

The LC is located in the rostral portion of the pons, right below the floor of the fourth ventricle [11]. In humans, it is formed by approx. 40,000–60,000 neurons that are organized in an elongated column, and it receives input from several brain regions, including the hypothalamus, raphe nuclei, and basal forebrain. The LC is richly innervated by noradrenergic, serotonergic, and cholinergic neurons, and it receives both excitatory and inhibitory inputs from these sources [12].

**Figure 2:**
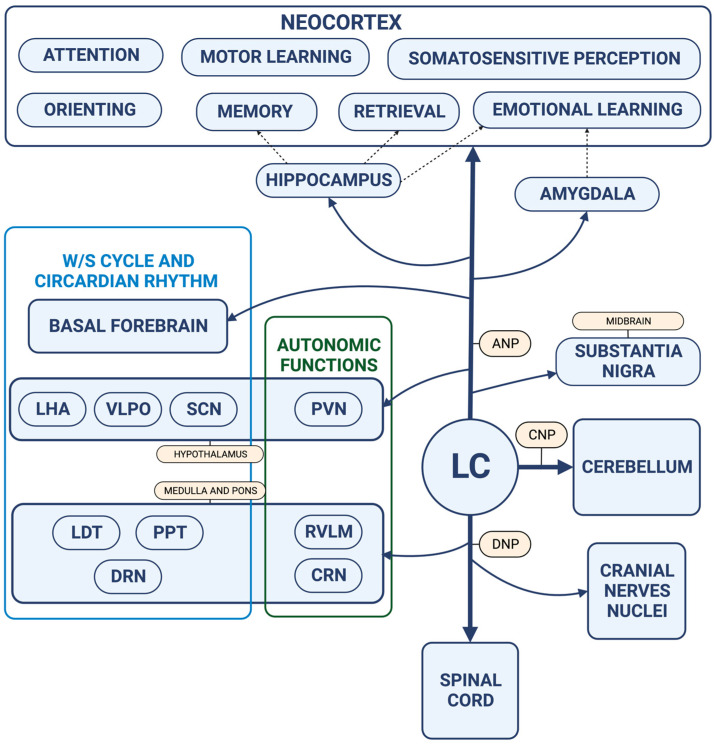
The LC provides a dense and widespread system of projections that virtually reaches the whole CNS. From the anatomical nucleus, NA fibers travels segmented into three pathways: an ascending pathway (ANP) through which the LC reaches the midbrain and the forebrain, a cerebellar pathway (CNP), and a descending pathway (DNP), which carries the NA fibers for the lower pons, the medulla, and the spinal cord. Through such anatomical connections, the LC enters the circuitries of the sleep/wake cycle and circadian rhythms regulatory nuclei and of autonomic system modulatory nuclei. Rostrally, the LC reaches the limbic system and the neocortex, taking part in the complex orchestration of cognitive and behavioral functions, such as attention and memory. (Created with Biorender.com, accessed on 15 March 2023). **Abbreviations:** ANP: Ascending Noradrenergic Pathway; CNP: cerebellar noradrenergic pathway; CNS: central nervous system; CRN: caudal raphe nuclei; DNP: descendent noradrenergic pathway; DRN: dorsal raphe nucleus; LC: locus coeruleus; LDT: lateral dorsal tegmental nucleus; LHA: lateral hypothalamic area; NA: noradrenaline; PPT: pedunculo-pontine tegmental nucleus; PVN: paraventricular nucleus; RVLM: rostral ventro-lateral medulla; SCN: suprachiasmatic nucleus; VLPO: ventro-lateral preoptic area; W/S: wake and sleep cycle.

As already said, the LC has a widespread and dense projection system that reaches virtually the whole CNS, including the cortex, hippocampus, amygdala, and spinal cord. Anatomical and functional connections between the LC and the other parts of the CNS are summarized in Figure 2. From the LC, NA fibers reach their target by forming three pathways: (i) an ascending path, which provides the NA innervation of the midbrain and forebrain; (ii) a cerebellar path, which projects to the cerebellum; and (iii) a descending path, which innervates the medulla and spinal cord [12]. LC axons release NA through two different mechanisms. One is the “classical” synaptic transmission, which takes place at the level of axon terminals, where NA is released from neurotransmitter vesicles into the synaptic cleft. This is called “wiring transmission” [13]. The other mechanism is typical of neurons belonging to the reticular formation and involves varicosities placed along the LC axons, which release NA in a paracrine fashion, thus modulating the activity of a broad area, irrespective of the cellular target. The latter is called “volume transmission” [13]. LC-NA exerts its several modulatory roles by acting on adrenoceptors, namely α_1_, α_2_, and β ones. A_1_ and β adrenoceptors are mainly post-synaptic and excitatory, while α_2_ are both pre- and post-synaptic and inhibitory [14]. However, the effect of LC-NA innervation is particularly complex and cannot be described only on the basis of targeted receptors. As an example, in sensory cortices, it produces a “signal-to-noise” reduction effect by promoting the activity of neurons specifically receiving the stimuli and inhibiting, at the same time, surrounding neurons [15]. In the prefrontal and temporal cortex, the LC has been shown to modulate the areas that play a critical role in the regulation of arousal and attention [16,17]. In the hippocampus, the LC modulates memory processes (both encoding and retrieval) [18,19], and in the amygdala, it takes part in emotional behavior [20].

Furthermore, LC-NA terminals influence those target areas not only by directly regulating the activity of neurons and interneurons [12], but also indirectly by modulating astrocytes functions and neurovascular response [21].

The LC also plays a role in the regulation of the sleep/wake cycle through a variety of mechanisms [22] and alterations in its activity have been implicated in sleep disorders [23]. The LC is a key station of the ascending reticular activating system (ARAS) [24], and it works as a wake-promoting nucleus [22]. It projects to the cholinergic neurons of the basal forebrain, the dopaminergic neurons of the ventral tegmental area and the orexinergic cells of the lateral hypothalamus, through whose stimulation it prompts cortical activity and EEG desynchronization [25]. GABAergic fibers from the anterior hypothalamus and GABAergic interneurons located next to the LC itself provide the inhibitory input, which reduces LC activity, in turn promoting sleep onset [23].

Furthermore, LC takes part in the modulation of circadian rhythms, projecting to the hypothalamus [26]. The connections between these two areas of the CNS also underlie the regulation of many autonomic and endocrine pathways, such as heart rate, blood pressure, and release of steroid hormones [27]. Finally, the LC also plays a role in the regulation of the stress response, and its activity is altered in stress-related disorders such as anxiety and depression [28,29,30].

## 4. LC Ontogenesis

LC originates within the rhombomere 1 of the developing hindbrain, the same part from which the cerebellum, the trigeminal, and the vestibular sensory column form [31,32]. In human embryogenesis, tyrosine hydroxylase (TH)- and dopamine-β-hydroxylase (DBH)- positive cells appear in this region approximately at the fifth gestational week [33]. Within the thirteenth week, noradrenergic innervation of the cortical plate is already detectable, expanding rostro-caudally from frontal to occipital regions [34]. Such an early origin might involve only the NA cells of the LC, as animal evidence suggests that they are already quite differentiated in fetuses [35] and that non-NA neurons might take longer to fully develop [36]. It is worth noting that the rapid ontogenesis of LC—as a neuronal cluster—is paralleled by the development and differentiation of its vascularization, with capillaries showing well-formed walls and organized blood–brain barrier features already in these early phases [35]. After birth, LC continues to develop and differentiate [37], with a progressive increase in the neuronal total count and separation into two clusters, one anterior and one posterior, possibly suggesting a topographical division of projection targets and functions [38]. At the same time, the NA forebrain innervation shows a huge growth, as witnessed by the increase in noradrenaline transporter (NAT) positivity, which rapidly reaches the levels observed in adulthood [39].

From a molecular perspective, LC ontogenesis is a very complex process, which requires the orchestration of many growth factors [40,41,42,43]. Bone morphogenetic proteins 2, 5, and 7; fibroblast growth factor 8; and the protein of the Wnt families have been found to stimulate the growth of TH-positive neurons of the LC [41]. A pivotal role seems to be played by the transcriptional factors Phox2a and Phox2b, which are mandatory for LC development in the context of the rhombomere 1 [43,44], promoting the expression of the DBH and NAT genes [42]. This molecular interplay is probably also modulated by target regions of LC themselves; in fact, in vitro evidence shows that in the co-culture of LC-NA and hippocampal cells, the latter promote the maturation of the formers [45,46].

## 5. Susceptibility of LC to Different Insults during Ontogenesis and Post-Natal Development

LC is particularly susceptible to damage caused by a variety of insults in the adult [47], and a similar sensitivity might be hypothesized to occur also during its fetal and post-gestational development [48,49,50,51].

Among the possible damaging causes, hypoxia has been studied more systematically and offers the largest body of evidence [48,49,50,51]. LC is particularly sensitive to hypoxic damage, which may lead to the morphological and cellular disruption of the nucleus during its ontogenesis and post-natal development [35]. In two neuropathological case series of sudden unexplained perinatal death, Lavezzi and colleagues showed a significant reduction in TH staining in the LC of human fetuses and newborns. [49,50]. The authors themselves suggested that the disruption of LC development might have contributed to the death of those infants, maybe hampering the normal functioning of the respiratory centers, in the regulation of which LC plays a key role [52]. More interestingly, Lavezzi and colleagues observed the occurrence of an association between maternal smoking and LC damage [49,50], suggesting hypoxia as a possible cause, mediated directly by the high level of CO in maternal blood and indirectly by the vasoconstrictive effect of nicotine [49].

In line with this data, another neuropathological study assessed LC integrity in infants who died in concomitance with hypoxic/ischemic injury [51]. The authors found that LC was the monoaminergic nucleus that suffered the worst damage from hypoxia, especially when prolonged and of moderate severity [51].

Beyond hypoxic/ischemic damage, the development of LC might also be compromised by other harmful substances or events, such as polluting compounds, which can be especially damaging during its ontogenesis [53]. For, bisphenol A (BPA), an industrial-derived xenoestrogen and common pollutant [54], has been associated with LC development disruption in a study in rodents [55]. Incidentally, BPA has also been linked to NdD, such as ASD [56,57].

Finally, some studies have associated maternal malnourishment with the alteration of NA innervation development and the impairment of frontal/attentive functions [58,59,60].

## 6. The Role of LC-NA Innervation in the Ontogenesis of CNS and Possible Consequences of Its Alteration

Beyond proper LC development and its modulation, the central NA system plays an important role in regulating and stimulating the ontogenesis of other areas of the CNS [61,62,63,64,65,66,67,68,69,70,71]. As mentioned, LC projections are already established during fetal life and can exert their effect on target areas [72].

Thus, the influence of the NA system on brain ontogenesis probably starts since the earliest stages of CNS development; NA neurons appear at the fifth gestational week in humans, and they can exert their modulation both in a paracrine and a synaptic fashion [72]. Studies performed in various animal species suggest that NA might promote the differentiation of neuronal progenitors from ectodermal cells [61,62]. In those studies, the authors administered exogenous NA or NA antagonists, observing an increase or a reduction, respectively, in neurogenesis [61,62]. Paralleling these findings, other studies found that the correct expression and functioning of NAT are crucial for the physiological neuronal differentiation in the developing brain, for both NA and non-NA neurons [63,64,65].

In the following stages of brain pre-natal development, NA might influence, together with other catecholamines, the differentiation and structural organization of the forebrain [66,67,68,69,70,71] and of the spinal cord [3]. The loss of NA might bear detrimental consequences on the development of the cortex; in primates, it has been observed that the administration of cocaine, which is responsible for transient NA depletion [68], alters the normal cortical development, reducing the count of neurons and disrupting the cortical architecture [69]. This functional evidence is then supported by histological studies, which revealed the occurrence of adrenoceptor and noradrenergic innervation in the periventricular zone [71] and in the developing cortical plate [70]; while in the first area, NA might exert a modulating effect of progenitor stem cells proliferation [71], in the cortical plate, it might play an active role in promoting cortex differentiation and formation of layers [70].

Finally, it is worth noting that NA keeps exerting its modulatory effects on neuronal development also in the post-natal period and even in adult life, although more weakly than during prenatal development [66]. Noradrenaline regulates synaptogenesis in the earliest phases of post-natal life, as has been observed in the visual cortex of rats [67]; in this study, the loss of NA innervation was linked to hyperactive synaptogenesis, leading to aberrant functioning of cortical networks [67]. In the adult, NA has been linked to hippocampal neurogenesis, even though the specific role in its regulation, i.e., whether it is promoting [73,74,75,76] or inhibiting [77], has not been clarified yet.

## 7. Preclinical Data Links NdD Symptoms with LC-NA System Dysfunction

Considering the early maturation of the LC and its role in brain development, it is not surprising that many researchers have hypothesized that alterations of the central noradrenergic system during critical periods might promote the onset of NdD. Despite its potential interest, only a few experimental studies on animal models of NdD have been performed and provide limited information. In particular, we included only three of those in this review, as they specifically focused on the LC-NA system in well-defined animal models of NdD [78,79,80]. The first two studies we selected focused on ADHD, particularly on the possible noradrenergic genesis of attention impairment and hyperactivity [78,79].

In particular, in 1995, de Villiers and colleagues tested the effect of brimonidine, an α_2_ adrenoceptor agonist, on spontaneously hypertensive rats (SHRs), which are considered an animal model for ADHD [78]. The authors based their experiment on the hypothesis that in SHR, there is a hyperactivation of the LC-NA system, which in turn inhibits dopamine release in the nucleus accumbens. Indeed, they found increased levels of NA and dopamine in the brains of SHRs, particularly in the prefrontal cortex and in the LC. However, brimonidine did not exert a specific effect on dopamine release and concentration. The authors suggested that a much more complex mechanism might underlie the interplay between these two biochemical systems, which could not be dissected using only their pharmacological approach. Nonetheless, they clearly showed that increased levels of NA are associated with ADHD-like symptoms [78]. In 2003, Jones and Hess performed another animal study, whose findings supported this evidence. They used the coloboma mouse model, which is considered another animal model for ADHD and spontaneously exhibits locomotor hyperactivity [79]. This abnormal behavior might be due to LC-NA system dysregulation, resulting from the reduction in the pre-synaptic protein SNAP-25, which is implied in NA release. Indeed, the authors lesioned the LC-NA system in these animals by DSP-4 administration and observed a dramatic reduction in locomotor hyperactivity. They interpreted that finding as proof supporting the hypothesis of the noradrenergic genesis of ADHD-like symptoms in those animals [79,80].

The third study we found was published in 2021 by Yin and colleagues, which explored the role of the LC-NA system in motor learning in an ADS animal model. Specifically, they studied the 16p11.2 deletion mouse model, which carries a genetic mutation commonly detected in ADS patients and found that the impairment of motor learning typically observed in this model was associated with abnormal functioning of the motor cortex network, whose activity was restored by pharmacogenetic activation of the LC-NA system. This resulted in the rescue of motor learning ability, and the authors interpreted this finding as evidence of the possible role of the LC-NA system in the pathophysiology of ADS [80].

## 8. Clinical Evidence of LC-NA System Involvement in NdD

The low number of preclinical studies parallels that of clinical investigations, which mainly focus on ASD and ADHD [4,81,82,83]. In ASD, an LC dysfunction has been proposed as a promoter of aberrant attentional function and altered neuromodulation [5,6]. As far as we know, LC integrity in vivo has been only tested by the indirect pupillometric assessment—a reliable surrogate marker of noradrenaline activity—which has indeed shown pupillary dilation is impaired in children with ASD [5] or through functional MRI [84], while no LC structural MRI studies have been performed thus far.

ASD includes a broad range of disorders mainly characterized by the impairment of social skills and cognitive alterations, such as repetitive behavior and attention deficit [7]. The proper control of attentive function requires two phases, namely alerting and orienting. Alerting is the ability to detect the occurrence of a new stimulus in the context of quiet or active arousal, while orienting is the ability to spatially localize the stimulus in the environment [5]. Both these steps are modulated by LC activity and are impaired in ASD children [6,17,85]. The interplay between NA activity, attention function, and ASD is supported by neuroimaging evidence. In 2021, Huang and colleagues studied the functional connectivity between the LC and the cortex in a group of ASD children and showed an impairment of the LC projection system toward several cortical areas, including the ones involved in somatosensory perception and attention [86]. Pupillometric studies further supported the impairment of the LC-NA system in these patients. ASD children show a larger resting-state pupil diameter (RSPD), which is linked to the tonic activity of LC, while abnormal diameter variations were observed after specific stimuli or tasks (task/stimulus-evoked pupil dilation response (EPDR)) [87,88,89,90,91], the latter parameter being associated with LC phasic activity [89]. The degrees of alteration of RSPD and EPDR were directly related one to another, supporting the hypothesis of a global dysfunction of the LC-NA system. Interestingly, EPDR was not merely reduced when compared to control patients, but showed an aberrant behavior, with lower response to social stimuli and higher to visual [92] or non-social ones [90,93,94].

An abnormal pupillary response was also found in ADHD patients, who suffer an attention impairment along with disorganization, and/or hyperactivity-impulsivity [8]. ADHD patients showed a strong increase in RSPD and dramatic alterations in EPDR, which could be almost suppressed [87,95], or were unusually hyperreactive to emotional stimuli [96]. Furthermore, an abnormal timeframe of pupil response was also observed in ADHD [97]. When performing this cognitive task, in normal conditions, the expected pupillary response is a mydriatic reaction (“cognitive shift”) followed by a reduction in diameter (“consecutive correction”). This progression of EPDR has been linked to specific patterns of LC functioning: a sudden increase in phasic activity due to the detection of the new stimulus (alerting) and then its reduction during the focusing phase (orienting). In children with ADHD, the pupil response follows an opposite behavior, with a myotic response to cognitive shifting and increased pupil diameter later during consecutive correction. This abnormal behavior appeared correlated to the performance at the Wisconsin Card Sorting Test, which is related to context formation and task switching among executive functions, suggesting that dysfunction of the LC-NA system may drive the impairment of executive functions in ADHD [97].

Data regarding the morphological features of LC in ADHD are few and of no univocal interpretation. One MRI study found a volumetric reduction in the pontine area containing the LC [84], while PET studies used the radioligand (S,S)-[18F]FMeNER-D2 to quantify the NAT, which did not show any significant difference with control subjects [98,99].

The strongest evidence in support of LC-NA system involvement in ADHD is derived from pharmacological data. Drugs exerting an NA-modulating effect are beneficial to ADHD patients. It is interesting to note that both adrenergic receptor agonists and antagonists have been proven to be effective, highlighting how an aberrant function, rather than an impaired or excessive one, could lead to NdD symptoms. However, it should be mentioned that these drugs show complex pharmacodynamics, acting upon many modulating systems at the same time [9]. In detail, atomoxetine, methylphenidate, and amphetamines increase NA synaptic concentration, while α2-Adrenergic receptor agonists such as guanfacine and clonidine reduce it. Nevertheless, they all have been shown to improve cognitive performance and ameliorate attentive deficits in ADHD, as well as attention focusing [9,10,100,101].

## 9. Potential Mechanisms Linking LC Impairment and Pathophysiology of NdD

**Figure 3:**
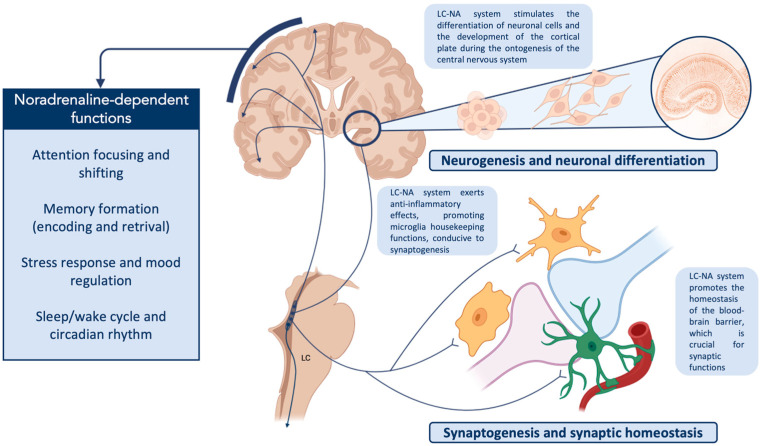
The LC-NA system modulates many neural networks (macroscale level) and regulates several homeostatic mechanisms (microscale level) of the central nervous system, many of which are involved in neurodevelopmental disorders. At the microscale level, LC-NA participates in synaptogenesis and synaptic plasticity, both as a neurotransmitter and through modulation of microglia and the promotion of blood–brain barrier integrity. At the same time, NA functions as a regulator of neurogenesis, a role that this system maintains during both pre-natal and post-natal life, up to adulthood. At the macroscale level, the LC-NA system regulates the activity of many neural networks, participating in the sleep/wake cycle and stress response. Moreover, it modulates cognitive functions such as attention and memory. Altered synaptogenesis and abnormal neurogenesis are suggested as possible pathogenetic mechanisms of neurodevelopmental disorders; in parallel, cognitive and behavioral alterations and mood and sleep disorders are common clinical features in these patients. Accordingly, a causal role of LC alteration during ontogeny and postnatal development has been hypothesized. (Created with Biorender.com, accessed on 15 March 2023). **Abbreviations:** LC: locus coeruleus; LC-NA: locus-coeruleus–noradrenergic System.

In the previous sections, we described the ontogenesis of the LC-NA system and its contribution to the development of the CNS, from the embryonal stage to the post-natal period, underscoring how this system is sensitive to damage or degeneration, and how its early impairment might cause morphological and functional alterations of other structures of the brain. Then, we summarized the available and most relevant pieces of evidence, clearly showing how the LC-NA system’s malfunctioning could be responsible for cognitive and behavioral alterations characterizing two of the most common NdDs, i.e., ASD and ADHD. Based on these premises, considering the growing amount of data about the role of LC in physiological functions and neurological disorders, we here made an attempt to propose a tentative hypothesis on the pathogenic and pathophysiological role of the LC-NA system in NdD. Figure 3 shows the physiological pathways modulated by the LC-NA system whose disruption might be involved in NdD.

As already pointed out, attentive functions are severely impaired in ASD and ADHD, and such impairments might be the clinical outcome of functional abnormalities occurring within the LC-NA system. Pupillometric studies show how children suffering from ASD and ADHD show a larger pupil diameter in resting conditions, and how it abnormally responds to environmental stimuli or cognitive tasks [5,8,87,88,89,90,91,92,93,94,95,96,97]. Consistently with our working hypothesis, the authors of those studies pose that such a pupillary behavior might reflect LC dysfunction in orchestrating attentive functions. Its wide projection system allows the LC neurons to reach the whole cortex and modulate its activity [19]. As shown in classical studies, LC provides such modulatory function by changing its firing rate; during NREM sleep, LC fires at its lowest frequencies and is completely silent during REM sleep, while its discharge rate increases in the transition from sleep to waking and from quiet waking to a novel or stressful environment. More specifically, LC firing could be divided into a tonic and a phasic discharge mode. The tonic activity depends on the vigilance condition (i.e., sleep or waking) and the outer environment (i.e., a quiet or stressful one). The phasic activity is elicited by the occurrence of new or unexpected stimuli, as well as by cognitive tasks that require attention focusing. These two firing patterns are related to each other; higher LC basal tonic firing caused by a stressful environment ensures an elevated state of alertness and improves the ability to detect any unexpected stimulus. In parallel, higher LC basal tonic firing limits the transition to phasic activity, thus hindering the ability to focus on a specific target. In contrast, the low tonic activity occurring under unstressful conditions allows for adequate LC phasic discharge, which will be finely tuned and corrected in relation to the stimulus or task on which the subject is focusing [16,17,85,102]. The unbalancing of this interplay is probably at the basis of attentive dysfunctions observed in NdD; in these patients, LC shows basal hyperactivity, with higher frequencies of tonic firing—as witnessed by the larger RSPD—which impairs the phasic firing and, consequently, the attention focusing or shifting [5,6].

It is worth noting that such an abnormal activity of LC might help to also explain other symptoms occurring in NdD. In children/young adults with NdDs, a high rate of non-NdD psychiatric disorders (affective, anxiety, and personality disorders), as well as sleep disturbances, has been acknowledged [103,104,105,106,107,108,109]. Such disturbances have already been associated with impaired LC function in patients with typical neurodevelopment [22,23,28,110]; hence, we may hypothesize NA dysfunction to also play a role in promoting these comorbidities in subjects with NdD. Indeed, in the case of ADHD, the abnormal LC hyperactivity might be linked to the increased level of anxiety and the shorter sleeping time observed in these patients [104,105].

Although the functional impairment of LC has been indirectly observed and suggested, morphological data about its involvement in NdDs are very limited. To our knowledge, no in vivo imaging studies specifically assessing the LC have been performed yet in ASD, and postmortem data are very limited. Among the latter ones, two studies did not find any pathological alterations in the LC of ASD subjects [81,82], while other pieces of evidence reported slight modification of the cytological architecture [4,83]. However, it should be mentioned that the former ones were conducted in very small cohorts of subjects and using incomplete neuropathological samples, while the latter ones were not designed to explore LC neuropathological features. In the case of ADHD, no neuropathological data are available, while in vivo imaging is limited to the study we reported in the previous section [84].

As said in Section 7, the lack of structural information is paralleled by the small amount of experimental data on animals. In fact, only a few studies explored the role of NA or LC in animal models of ADHD [78,79] or ASD [80] and the causal link between NdD and LC is still unclear.

Thus, we used also another approach to further dissect the potential interaction between LC impairment and NdD pathogenesis, i.e., we explored whether those genes associated with ADHD and ASD thus far are also involved in the modulation of the NA system.

We found that some genetic loci that have been associated with NdD are related to LC-NA system activity and physiology. The most relevant of these is SLC6A2, which encodes for NAT and has been proposed as a susceptibility gene for ADHD [111,112] but not for ASD [113]. The gene family SHANK, encoding for post-synaptic proteins whose role is to join neurotransmitter receptors to the cell cytoskeleton, has been associated with ASD [114]. The three different SHANK genes can produce multiple protein isoforms that are differentially expressed according to developmental stages, cell types, and brain regions. Mutations in SHANK genes can exert a pathogenic role in children with ASD [115,116]. Interestingly, one study showed that the lack of the protein SHANK1 is responsible for the loss of the hyperactivating effect of amphetamine in a transgenic mouse model, a pharmacological effect that is considered to be at least in part mediated by NA [117]. NOS3 has also been genetically linked to ASD [118]. NOS3 encodes for the endothelial nitric oxide synthetase, which plays a major role in brain homeostasis [119], and, as some studies suggest, also modulates LC activity [120,121]. Finally, the gene MECP2 has been linked to ASD-like disorder in the context of Rett syndrome, a X-linked neurodevelopmental disease [122,123,124]. It has been shown that, in mice lacking the MECP2 gene, LC neurons exhibit several abnormal electrophysiological properties, pointing toward an LC-NA system dysfunction in this disease [125].

Besides genetic association, also environmental factors that have been linked to NdD might affect LC, in particular during its fetal development. Heavy metals, maternal obesity or eating disorders, smoking, and imbalance of sexual hormones are considered risk factors for the development of ASD [126] and ADHD [127], and all of them are known to be toxic or, at least, to impair the LC proper function [49,50,55,57,128,129,130,131,132,133].

The impairment of LC-NA system might not only contribute to functional impairment associated with NdD, but also to the pathogenesis of NdD. In other words, the dysfunction of LC might not only result in cognitive and behavioral alterations, but it might also directly affect brain homeostatic and cellular mechanisms, thus taking an active part in NdD pathology.

Synaptogenesis is severely impaired in ASD and ADHD, and this is thought to be one of the main pathogenic mechanisms underlying these disorders [134,135,136,137]. As already described above, LC plays a crucial role in synaptogenesis, both during brain development and in adulthood [67,138,139,140,141]. It might be hypothesized that the LC dysfunction occurring in NdD might contribute to the synaptic impairment observed in these disorders, via microglial-related mechanisms. Indeed, LC is a powerful modulator of microglial cell activity [142,143] as it exerts anti-neuroinflammatory effects, keeping microglial cells in a quiescent state so that LC impairment has been specifically associated with aberrant microglial functioning [143]. In turn, microglial and astroglial alterations have been suggested as possible mechanisms through which the impairment of synaptogenesis occurs in NdD [144,145,146,147,148]. Glial cell alterations might also represent a link between synaptic impairment and alterations in NdD by affecting the integrity of the blood–brain barrier (BBB), which represents another key morpho-functional unit that has been shown to be altered in ASD in parallel with increased neuroinflammation [149]. In line with this, LC-NA is key for BBB integrity [150], and thus LC alteration might contribute to ASD pathogenesis also through an alteration of this path. Indeed, it is likely that the LC-NA system plays a crucial role in the regulation of the so-called neurovascular unit, a morpho-functional complex constituted by capillaries, astrocytes, and microglial cells [21]. In such a context, a hypothetical contribution to NdD pathogenesis might pass through the alteration of the regulating effect the LC exerts on the neurovascular unit.

## 10. Future Perspectives

The literature data we reported in the previous paragraphs clearly illustrate how the LC-NA system might be involved in NdD, particularly considering ADS and ADHD. However, we are tempted to formulate further hypotheses on this topic based on the same evidence.

The high sensitivity of LC to hypoxic damage [35,48,51] makes it an intriguing target for future studies on infantile cerebral palsy, one of the most important causes of childhood disability, which is often caused by ischemic injury [151]. Even though hypoxic damage might be sufficient per se to explain the occurrence of both the forebrain and the LC injury, it should be noted that the LC-NA system exerts a protective role on ischemic damage, promoting the correct functioning of the neurovascular unit [21]. In line with this, a possible contribution of LC disruption in the pathogenesis of infantile cerebral palsy might be hypothesized, and it may be worth future investigations.

Further expanding such speculation, the impairment of LC-NA system might be suspected as a culprit also in another group of childhood disorders, namely epilepsy. The role of the LC-NA system in adulthood epilepsy is well-known and documented [138,152,153]; the experimental lesion of LC has been associated with a reduction in seizure threshold [153], and the loss of LC proper functioning in vivo has been suggested as a possible pathogenic mechanism [154]. Thus, exploring the involvement of the LC-NA system in childhood epilepsy would represent the extension of what is already known in adulthood, which may represent the theoretical basis for clinical and experimental investigations.

In order to perform those future studies, appropriate experimental and diagnostic tools will be needed.

Since animal models have been used extensively to test the role of LC lesions in other neurological disorders (especially neurodegeneration [155,156,157]), hopefully existing experimental models of LC lesioning might be systematically applied also to models of NdD to test this link, dissect the potential mechanisms through which LC impairment might contribute to their pathogenesis, and, eventually, evaluate beneficial effects of ad hoc developed NA-targeting therapeutic approaches.

Concerning clinical research, the recent development of a reliable neuroimaging tool to visualize the LC in vivo and assess its integrity, the LC Magnetic Resonance Imaging (LC-MRI) [1] might offer a chance to explore the alterations this nucleus might suffer from in NdD. Even though this tool has been used only in adults or elderlies till now, LC-MRI may also be applied to childhood disorders. A variety of technical and radiological features support the applicability and informativeness of this technique.

The small and highly variable size of LC may raise questions about the actual applicability of LC-MRI in children, whose brains are smaller than those of adults. Moreover, the LC of children contains only a small amount of neuromelanin (NM), a pigmented by-product of NA [158], which by binding metal ions and acquiring paramagnetic properties might represent a source of LC signal at MRI [159]. However, it should be mentioned that some studies have been successfully performed on very young subjects [160,161,162]. Furthermore, a growing amount of evidence is proving that NM might not be the real source of LC hyperintensity in MRI, and it might be explained in light of the so-called “Magnetization Transfer” effect [163,164,165]. The latter is a particular MRI phenomenon that takes place in small brain structures, densely packed within white matter bundles, as is the case in LC [166,167]. According to this hypothesis, the low amount of NM occurring in child LC would not hamper the usefulness of LC-MRI in children. In support of this, Watanabe and colleagues performed a study in 2019 in mice knocked out for the gene DBH, thus synthesizing very low amounts of NA and of NM. In MRI, the LC signal was not affected, and the authors suggested this proved that NM is not its real source [168]. The latter data strongly support the rationale of the possible applicability of LC-MRI also to children.

Concerning the informativeness of LC-MRI, another potential issue one might face is that in NdD, LC could undergo a heterogenous variety of alterations significantly different from the degenerative phenomena occurring in elderly patients [169,170]. Even though this might be the case of hypoxic/ischemic injuries, in which a reduction in LC signal might be detected, the data we reported above suggest that in NdD, the LC-NA system shows an aberrant functioning, which might not be necessarily paralleled by structural alterations of the LC itself. However, it is worth noting that in studies performed in adults, LC-MRI signal has been shown to be associated with LC-related cognitive and autonomic functions, even in the absence of brain pathology [160,161,162,171,172]. Furthermore, in a very interesting recent pre-print, Bachman and colleagues found that LC signal can vary in follow-up assessments, after autonomic biofeedback training [173]. Those studies suggest that LC-MRI parameters are associated with LC functioning and plasticity, even in the absence of morphological alterations, which might be a potential scenario occurring in NdD. In line with this, using this tool also in NdD children may be as informative as it is in adults and elderlies.

## 11. Conclusions

In this paper, we reviewed the available evidence about the interplay between the LC-NA system and NdD; even though the amount of data is not exhaustive, in our opinion, it is still sufficient to draw a noradrenergic contribution of developmental disorders. The early origin of the LC during ontogenesis, its important role in modulating brain development, and its sensitivity to perinatal damage make this nucleus an interesting spot on which future research might focus, pointing toward a better understanding of the molecular and pathological mechanisms underlying it.

In conclusion, in the enormous number of polygenic and multifactorial combinations of risk factors and pathological pathways that might lead to the occurrence of NdD, the LC-NA system may represent a common pathophysiological and pathogenic mechanism and a potential target for symptomatic and disease-modifying drugs.

## Data Availability

Not applicable.
